# App-based support for breast cancer patients to reduce psychological distress during therapy and survivorship – a multicentric randomized controlled trial

**DOI:** 10.3389/fonc.2024.1354377

**Published:** 2024-04-18

**Authors:** Josefine Wolff, Svenja Seidel, Pia Wuelfing, Michael Patrick Lux, Christine zu Eulenburg, Martin Smollich, Freerk Baumann, Stephan Seitz, Sherko Kuemmel, Marc Thill, Joke Tio, Michael Braun, Hannah Hollaender, Angenla Seitz, Felicitas Horn, Nadia Harbeck, Rachel Wuerstlein

**Affiliations:** ^1^ Breast Center, Department of Gynecology and Obstetrics, and Comprehensive Cancer Center Ludwig-Maximilian University Munich (LMU) University Hospital, Munich, Germany; ^2^ Department Clinical Research, PINK! Gegen Brustkrebs GmbH, Hamburg, Germany; ^3^ Department of Gynecology and Obstetrics, Frauenklinik St. Louise and St. Josefs-Krankenhaus, St. Vincenz Klinik GmbH, Paderborn, Germany; ^4^ Department for Medical Biometry and Epidemiology, University Medical Center Hamburg-Eppendorf, Hamburg, Germany; ^5^ Institute of Nutritional Medicine, University Hospital Schleswig-Holstein, Luebeck, Germany; ^6^ Department I of Internal Medicine, University of Cologne, Cologne, Germany; ^7^ Department of Obstetrics and Gynecology, Caritas Hospital St. Josef, University of Regensburg, Regensburg, Germany; ^8^ Breast Unit, Kliniken Essen-Mitte, Essen, Germany; ^9^ Markus Hospital, Breast Center, Frankfurt, Germany; ^10^ Department of Gynecology and Obstetrics, University Hospital of Münster, Münster, Germany; ^11^ Department of Gynecology, Breast Center, Red Cross Hospital, Munich, Germany

**Keywords:** breast cancer, psychological distress, app-based coaching, depression, digital intervention, supportive care in cancer

## Abstract

**Introduction:**

The negative impact of unmanaged psychological distress on quality of life and outcome in breast cancer survivors has been demonstrated. Fortunately, studies indicate that distress can effectively be addressed and even prevented using evidence-based interventions. In Germany prescription-based mobile health apps, known as DiGAs (digital health applications), that are fully reimbursed by health insurances, were introduced in 2020. In this study, the effectiveness of an approved breast cancer DiGA was investigated: The personalized coaching app PINK! Coach supports and accompanies breast cancer patients during therapy and follow-up.

**Methods:**

PINK! Coach was specifically designed for breast cancer (BC) patients from the day of diagnosis to the time of Follow-up (aftercare). The app offers individualized, evidence-based therapy and side-effect management, mindfulness-based stress reduction, nutritional and psychological education, physical activity tracking, and motivational exercises to implement lifestyle changes sustainably in daily routine. A prospective, intraindividual RCT (DRKS00028699) was performed with n = 434 patients recruited in 7 German breast cancer centers from September 2022 until January 2023. Patients with BC were included independent of their stage of diseases, type of therapy and molecular characteristics of the tumor. Patients were randomized into one of two groups: The intervention group got access to PINK! over 12 weeks; the control group served as a waiting-list comparison to “standard of care.” The primary endpoint was psychological distress objectified by means of Patient Health Questionnaire-9 (PHQ-9). Subgroups were defined to investigate the app’s effect on several patient groups such as MBC vs. EBC patients, patients on therapy vs. in aftercare, patients who received a chemotherapy vs. patients who did not.

**Results:**

Efficacy analysis of the primary endpoint revealed a significant reduction in psychological distress (least squares estimate -1.62, 95% confidence interval [1.03; 2.21]; p<0.001) among intervention group patients from baseline to T3 vs, control group. Subgroup analysis also suggested improvements across all clinical situations.

**Conclusion:**

Patients with breast cancer suffer from psychological problems including anxiety and depression during and after therapy. Personalized, supportive care with the app PINK! Coach turned out as a promising opportunity to significantly improve psychological distress in a convenient, accessible, and low-threshold manner for breast cancer patients independent of their stage of disease (EBC/MBC), therapy phase (aftercare or therapy) or therapy itself (chemotherapy/other therapy options). The app is routinely available in Germany as a DiGA.

Clinical Trial Registration: DRKS Trial Registry (DRKS00028699).

## 1 Introduction

Breast cancer patients face an increased risk of experiencing psychiatric disorders, including depression and anxiety (1). The link between depression and anxiety status and cancer outcomes has been well investigated. Psychological distress is related to higher cancer-specific mortality and poorer cancer survival in patients with breast cancer (2–4). Both, low levels of psychological distress and low fatigue are independently correlated with longer periods of recurrence-free survival and overall survival (3, 4). In addition, psychological distress is also associated with a lower quality of life and an increased incidence of side effects (5, 6).

Overall, the negative impact of unmanaged psychological distress on breast cancer patients has numerous consequences for those affected, in terms of their treatment outcomes, survival, recurrence, as well as their daily life after acute therapy and long-term psychosocial well-being (3–8). Fortunately, studies indicate that distress can be effectively addressed and even prevented using digital, evidence-based interventions (9–14). Existing data also indicate that psychological interventions have the potential to impact neuroendocrine factors such as cortisol levels, as well as immune function markers, particularly lymphocyte proliferation and the production of TH1 cytokines. Psychological interventions can have a detrimental impact on various biological processes relevant to breast cancer. Initiation and progression of cancer involve a complex series of steps, including environmental exposures, genetic alterations, evasion of apoptosis, cell proliferation, evasion of immune surveillance, angiogenesis, and metastasis. Emerging evidence suggests that psychosocial stress can influence the trajectory of the disease at multiple stages within this process (15–18). Offering digital psychological support to breast cancer patients could have clinically meaningful psychological as well as biological effects.

With an annual incidence of 70.000 new cases in Germany alone, and a substantial number of survivors, the healthcare system is struggling to meet the increasing demand for supportive care and psycho-oncological support during and after initial breast cancer treatment. This challenge is particularly pronounced in rural areas where the access to care is noticeably lower compared to urban regions (19, 20). In acute cases, patients in most clinics have the opportunity to schedule a short-term appointment with a psycho-oncologist. However, psychotherapeutic support during and after therapy comes with long waiting times. Currently, in Germany, patients have to wait around 4 months for an outpatient psychotherapy spot.^1^

To address this gap in healthcare provision and improve support for breast cancer patients and survivors across all stages of treatment, digital app-based solutions present a promising and cost-effective opportunity. Research indicates that eHealth tools are emerging as effective platforms for delivering lifestyle interventions during breast cancer care (21, 22). Given the prolonged survival and long-term follow-up required for both early-stage and some metastatic breast cancer patients, studies emphasize the importance of providing evidence-based information on nutritional strategies, incorporating physical activity into daily routines, managing treatment side effects, and offering mental health coaching (23–26).

Due to multimodal therapeutic concepts for BC patients, especially EBC patients, treatments lead to full recovery in more than 70-80%. The burden of survivorship can indeed be overwhelming. Breast cancer survivors face a wide range of physiological and psychosocial challenges, often dealing with late and long-term effects from intensive treatment therapies. Despite these difficulties, breast cancer survivors express a strong desire to actively manage their health. This leads to a wide range of app-based support offerings for breast cancer patients worldwide. Mobile health (mHealth) apps can play a crucial role in addressing this need by providing patients with a dynamic platform to continuously monitor and track symptoms over time. These apps allow survivors to interact with peers for support and discussions on survivorship topics, offer resources for caregivers, send reminders for medication or follow-up appointments, and provide relevant education on managing health concerns during survivorship. Nevertheless, current studies indicate the need of criteria for end users and clinicians to help choose the right apps for better clinical outcomes (27–29).

The PINK! Coach App offers individualized, evidence-based therapy and side-effect management, mindfulness-based stress reduction, nutritional and psychological education, physical activity tracking and motivational exercises to implement lifestyle changes sustainably in daily routine of breast cancer patients. PINK! aims to support patients with breast cancer in every stage of disease and therapy in order to empower patients to take an active role during their therapy and in aftercare independent of factors such as stage of disease, type of therapy, age, place of residence or the clinic the patient is being treated or was treated. Using PINK! offers patients personalized, time- and location-independent coaching (14).

As a DiGA (German: “Digitale Gesundheitsanwendung”, Engl.: “Digital health application”) PINK! Coach is one of 47 prescription-based mobile health apps^2^ in Germany that are fully reimbursed by health insurances. To become a DiGA, mHealth applications undergo a rigorous certification process that includes providing scientific evidence of efficacy through clinical trials. Other certification requirements encompass safety, functional capability, quality, interoperability, data protection, and data security. As a result, some of the main barriers to patient adoption, such as high costs, lack of integration with current standards of care, and concerns about quality, are addressed within the German system, thus making it unique from an international perspective (30).

The preceding pilot study with the self-management app PINK! demonstrated effective reduction in psychological distress and fatigue among breast cancer patients and survivors. Our study revealed that participants who had access to the app and received individual coaching for 12 weeks experienced significant improvements in their levels of psychological distress and fatigue symptoms on average. Additionally, the usage of the app also led to an increase in physical activity levels within the 12-week period. Particularly noteworthy were the positive effects observed in participants who extensively utilized the app, defined as those who used it for at least 200 minutes over the 12 weeks (14).

The present study was designed to demonstrate the medical benefits and positive care effects of the app in terms of psychological distress among breast cancer patients in a multicentric setting to substantiate and validate the results of the pilot study. The study design of this research was further specified to investigate more precise insights into the mechanisms of action of the app on all breast cancer patients. Due to the numerous treatment options available for breast cancer, the individual situations of the patients vary significantly. As a result, the app must also be tailored to each individual to provide comprehensive support to all patients. We hypothesized that PINK! Coach empowers breast cancer patients at every stage of the disease to enhance their daily routines and lifestyle, thereby enabling them to live a healthier life in a sustainable manner and consequently reduce psychological distress.

## 2 Methods

The PINK! Coach App study was approved by the Medical Ethical Committee of the LMU University of Munich, Germany on 21.07.2022 (Reference number: 22-0498) Furthermore, the Medical Ethical Committees of all clinics that participated in this multicenter RCT approved of this study. The trial has been registered prospectively in the DRKS Trial Registry (DRKS00028699).

### 2.1 Study design

In this multicenter RCT, all breast cancer patients were recruited and randomly assigned to either intervention group (IG) or control (CG). CG served as a waiting list comparison to the “standard of care”. IG immediately received access to PINK! Coach on top of the “standard of care” while CG received access to PINK! Coach after 12 weeks. Given the design of the intervention, participants were not blinded to group assignment. A 1:1 randomization was performed with a computer-generated sequence. The participating investigators were blinded to the group allocation and did not have access to the collected data. Data were collected at baseline (before randomization), after 4 weeks (T1), after 8 weeks (T2) and after 12 weeks (T3) which was the primary endpoint of this study. To investigate long-term effects of the PINK! Coach App, a follow-up 6 and 12 months after baseline is currently being conducted.

### 2.2 Recruitment and inclusion criteria

Patients were recruited at 7 German Breast cancer Centers with the Breast Center of the LMU University Hospital in Munich as the Principal Investigator. Patients had to meet the following inclusion criteria: They had to be at least 18 years old, German-speaking, diagnosed with histologically confirmed breast cancer, own a smartphone, have an email address, and be willing to use an app as a therapy companion. In addition, the diagnosis of the initial occurrence or recurrence should have been made within the past 12 months or up to 12 months after the surgery. Furthermore, patients should either be undergoing therapy for at least 12 weeks or have been discharged to post-treatment follow-up (aftercare) at the time of recruitment. For all patients with metastatic breast cancer (MBC), the therapy status (first-line or second-line) is irrelevant, as all MBC patients were assigned to the treatment group.

Patients were identified, informed and included in the study at all participating breast cancer centers. All patients in this study signed a written informed consent. After baseline documentation and the patients first questionnaire, they were randomized to IG and CG. The following inclusion and exclusion criteria were defined:

- they had to be at least 18 years old,
- German-speaking,
- diagnosed with histologically confirmed breast cancer,
- own a smartphone,
- have an email address.
- and be willing to use an app as a therapy companion.

### 2.3 Intervention

PINK! Coach was designed specifically for early-stage and metastatic breast cancer (BC) patients from the day of diagnosis to the time of aftercare. The app offers individualized, evidence-based therapy and side-effect management, mindfulness-based stress reduction, nutritional and psychological education, physical activity tracking, and motivational exercises to implement lifestyle changes sustainably in daily routine. PINK! Coach offers multimodal content in 3 categories: nutrition, physical activity, and mental health. Content is provided as articles, videos, podcasts and daily goals to achieve. Those daily goals are steps counts, nutritional habits, physical exercises, MBSR exercises and more. The patients decide themselves if and how many goals they try to achieve each day. The goal is to motivate patients to start changing daily lifestyle habits. All information is evidence-based, validated, and permanently updated based on recent research results and current German Breast Cancer Guidelines (AGO recommendations; S3 guidelines) by the PINK! research expert board. To maintain and strengthen the motivation for change in patients, PINK! Coach incorporates various motivating elements. One element is the automated and pseudo-individualized coaching delivered contextually through push notifications. In order to boost motivation and performance, users also receive success messages upon achieving goals and can continually track their success statistics.

Through an automated chatbot, patients have the opportunity to gather information about the side effects of medication-based tumor therapies (chemotherapy, immunotherapy, antibody therapy, hormone therapy), radiation therapy, and breast cancer surgeries. Assuming that the entered symptoms are related to the side effects of the medications, the chatbot provides recommendations, primarily focusing on self-help tips (i.e., the application of conventional home remedies and behaviors). The symptoms are differentiated according to CTCAE criteria. In case of more severe symptoms the chatbot directly advises contacting the doctor without providing further self-help recommendations. The goal of the chatbot is to provide patients with confidence regarding the occurrence, intensity, and management of side effects, thereby reducing psychological distress.

The personalization of content is achieved through regular check-ups. Initially, these check-ups are requested for download, and then they are repeated at 2-week or 4-week intervals. The check-ups include questions about tumor biology and the status of therapy. Additionally, validated questionnaires about therapy-related side effects and psychological stress are administered. General information such as the individual’s professional situation is also taken into account.

As part of the change process, PINK! Coach guides patients into self-observation, where they consciously perceive their nutrition and physical activity. This is done through nutrition and exercise tracking. The results are analyzed and presented on a weekly and monthly basis, making the impact of individual decisions visible for success. Another important aspect is the setting of (intermediate) goals. The patient is supported in selecting possible goals appropriate for the specific time. The course contents build on each other, leading gradually to a successful adaptation of habits. The mental health section includes a 12-week course based on elements of cognitive-behavioral therapy aimed at reducing mental stress, anxiety, and other related effects.

**Figure 1:**
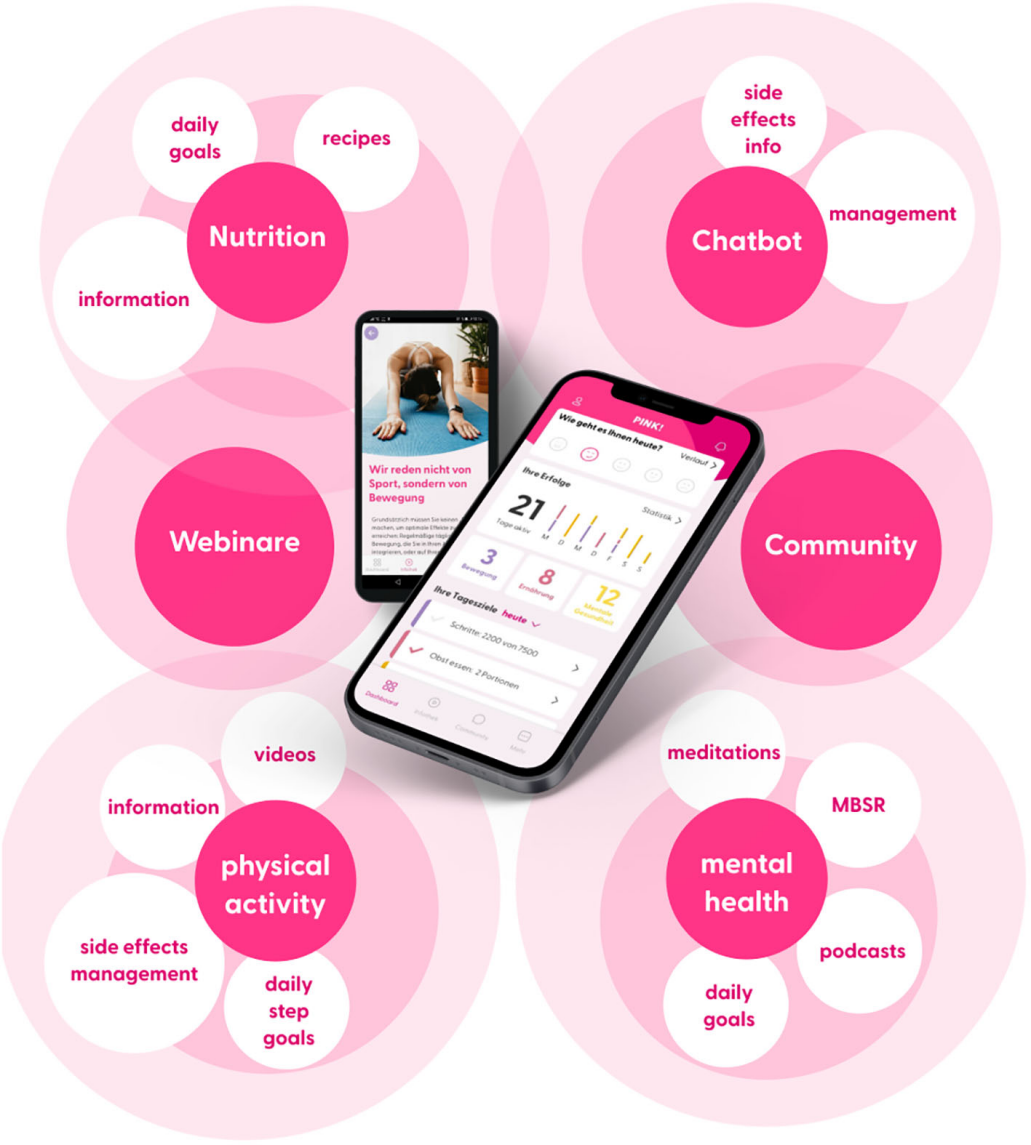
PINK! Coach App functionalities.

These multimodal features, shown in **Figure 1**, of the PINK! Coach app aim to individually address the complex interaction of psychological stress, quality of life, side effects, and personal aspects, in order to help each patient to improve their lifestyle in a tailored manner. PINK! Coach was developed by PINK! gegen Brustkrebs GmbH.

### 2.4 Objective and outcome measures

The primary objective of this study was to test whether using PINK! Coach App over 12 weeks during therapy or in aftercare leads to a significant reduction of psychological distress in patients with early-stage and metastatic breast cancer compared to control group that provided comparison to “standard of care” as waiting list.

The PHQ-9 is a versatile tool used for screening, diagnosing, monitoring, and assessing the severity of depression. It consists of nine items, and the total score is calculated by assigning scores of 0, 1, 2, and 3 to the response categories of “not at all,” “several days,” “more than half the days,” and “nearly every day,” respectively. The PHQ-9 total score can range from 0 to 27, obtained by summing up the scores of all nine items. Specific cut-off points are used to classify the severity of depression: scores of 5, 10, 15, and 20 represent mild, moderate, moderately severe, and severe depression, respectively.

The total scores of the PHQ-9 allow for the classification of the severity of depression. This helps assess the clinical relevance of changes in the score over a specific period of time. The classification of the severity of the PHQ-9 score thus provides a practical means of evaluating progress or treatment efficacy in depression. The following **Table 1** displays the clinical evaluation of the PHQ-9.

**Table 1:** Clinical evaluation of PHQ-9 score points in 5 groups of depression symptoms.

Scorepoints PHQ-9	Clinical evaluation
**0-4**	Minimal or no depression: The patient exhibits only a few depressive symptoms or no depressive symptoms at all.
**5-9**	Mild depression: The patient exhibits mild depressive symptoms that do not significantly impair daily functioning.
**10-14**	Moderate depression: The patient exhibits moderate depressive symptoms that may cause some impairment in daily functioning.
**15-19**	Moderately severe depression: The patient exhibits pronounced depressive symptoms that noticeably impair daily functioning.
**>20**	Severe depression: The patient exhibits severe depressive symptoms that significantly impair daily functioning and may require intensive treatment.

The transition to another group can be considered a clinically relevant change and is highly likely to have a noticeable impact on the patient (31).

### 2.5 Statistical analysis

The Full Analysis Set (FAS) contained both adherent and non-adherent patients, who were randomized and had at least the baseline PHQ-9 assessment. As sensitivity analysis, we analyzed changes in scores for only adherent patients (per protocol) which were all patients that finished all questionnaires (Baseline, T1, T2, and T3). Significance level was set at *p* ≤.05. The demographic, medical history and outcome variables were described using frequency and descriptive statistics. Analyses were performed using STATA Version 16.0.

The primary endpoint was the difference in changes from baseline in the PHQ-9 total score at T3 (12 weeks). As primary analysis, a linear mixed model (random intercept model) was applied, adjusting for baseline PHQ-9 and therapy status (therapy or aftercare). For sensitivity analysis, different imputation algorithms were used to deal with missing values at post-assessment. Subgroups according to therapy group (therapy or aftercare), chemotherapy (yes or no) and stage (EBC or MBC/recurrent disease) were analyzed similar to the primary analysis.

An analysis of response was applied using a generalized linear mixed model similar to the primary analysis, with response as a dichotomous endpoint. Standardized effect sizes (Cohen’s d) were calculated.

The PINK! Coach App study has been approved by the Medical Ethical Committee of the LMU University of Munich, Germany on 21.07.2022 (Reference number: 22-0498) Furthermore, the Medical Ethical Committees of all clinics that participated in this multicenter RCT approved of this study. The trial has been registered prospectively in the DRKS Trial Registry (DRKS00028699).

To assess the clinical relevance of the determined results, an attempt was made to determine the Minimal Clinically Important Difference (MCID) in addition to calculating effect sizes using Cohen’s method. The Minimal Clinically Important Difference of the PHQ-9 has not yet been determined for a cohort of breast cancer patients. There are no publications on this topic in the known databases. However, there are publications on the MCID of the PHQ-9 in other patient cohorts with different diagnoses. Therefore, it is currently not possible to directly compare our results with the data published so far. Nevertheless, responder analyses were conducted to assess clinical relevance.

## 3 Results

**Figure 2:**
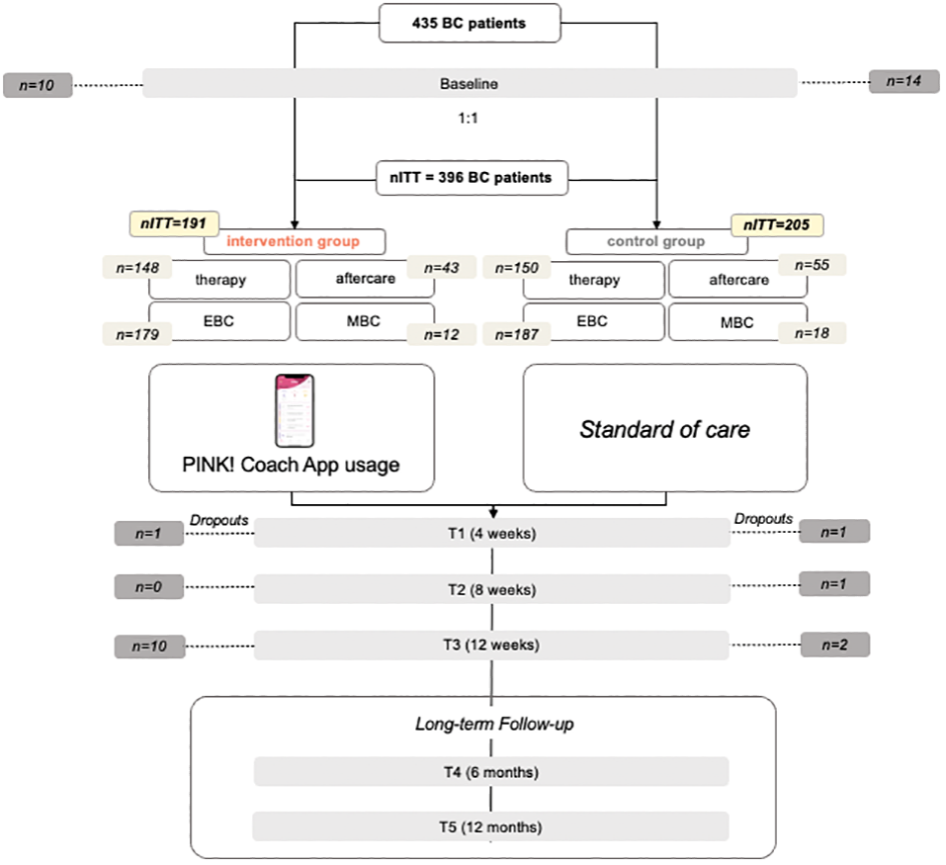
Study flow chart with number of patients, EBC/MBC, therapy/aftercare and dropouts.

From September 2022 until January 2023, a total of 435 patients met the inclusion criteria and signed the informed consent, answered the baseline questionnaire, and were randomized to one of the study groups IG or CG. 191 patients were randomized to the IG, 205 to the CG whereby 298 were in therapy and 98 in aftercare. Over 12 weeks there were 39 dropouts in total, with 21 in the IG and 18 in the CG which corresponds to 9.0% dropout rate. 50.3% of CG and 49.7% of IG received a chemotherapy. **Figure 2** displays the recruitment flowchart.

The data show a homogeneous distribution of patients in the IG and CG with respect to the recorded variables. The collective is heterogeneous due to broad inclusion criteria, but the distribution across the study groups is homogeneous.

**Table 2** displays the distribution of tumor biological characteristics of the entire collective in the IG and CG as well as in the subgroups of therapy and aftercare patients divided by EBC and MBC. Patients with TNBC (triple-negative breast cancer) or HER2-positive breast cancer are approximately equally distributed across both groups. Patients with hormone receptor-positive tumors are evenly distributed across both groups.

**Table 2:** Patient characteristics ITT collective EBC/MBC.

	Parameter	Value	Intervention	Control	Therapy	aftercare
**EBC**	*Number of patients*	*n (%)*	182 *(100.0)*	188 *(100.0)*	272 *(100.0)*	98 *(100.0)*
	*Median Age*		52.0	52.0	52.0	53.0
	*HR+ HER2 neg.*	*n (%)*	106 *(58.2)*	103 *(54.8)*	139 *(51.1)*	70 *(71.4)*
	*HER2 pos.*	*n (%)*	40 *(22.0)*	44 *(23.4)*	66 *(24.3)*	18 *(18.4)*
	*TNBC*	*n (%)*	36 *(19.8)*	41 *(21.8)*	67 *(24.6)*	10 *(10.2)*
	*CHT*	*%*	*51.2*	*44.9*	*58.3*	*20.9*
**MBC**	*Number of patients*	*n (%)*	9 *(5.6)*	17 *(9.5)*	26 (8.7)	0 (0.0)
	*Median Age*		52.5	54.5	52.5	–
	*HR+ HER2 neg.*	*n (%)*	2 *(22.2)*	2 *(11.8)*	4 *(15.4)*	–
	*HER2 pos.*	*n (%)*	6 *(66.6)*	12 *(70.6)*	18 *(69.2)*	–
	*TNBC*	*n (%)*	1 *(11.1)*	3 *(17.6)*	4 *(15.4)*	–
	*CHT*	*%*	33.3	47.0	42.3	–
***Total***	*Number of patients*	396 (100.0)				
	*Median Age*	52.0				
	*HR+ HER2 neg.*	*n*	209			
	*HER2 pos.*	*n*	84			
	*TNBC*	*n*	77			
	*CHT*	*%*	48.1			

The median age is 52 years. The median age of the IG and CG differs marginally. The variances in both groups are nearly equal.

### 3.1 PHQ-9 overall IG vs. CG

The primary endpoint was the difference in PHQ-9 reduction at T3 between IG and CG. In the IG, the PHQ-9 total score showed a reduction (delta) from baseline to T3 (12 weeks) of -1.5 score points (least squares estimate difference between IG and CG -1.52; 95% confidence interval (CI) [–1,91, –1,07] The calculation of difference values (Deltas) was not based on the mean values at different time points but rather as the mean of the difference values for each pair of values at the respective time points.

**Figure 3:**
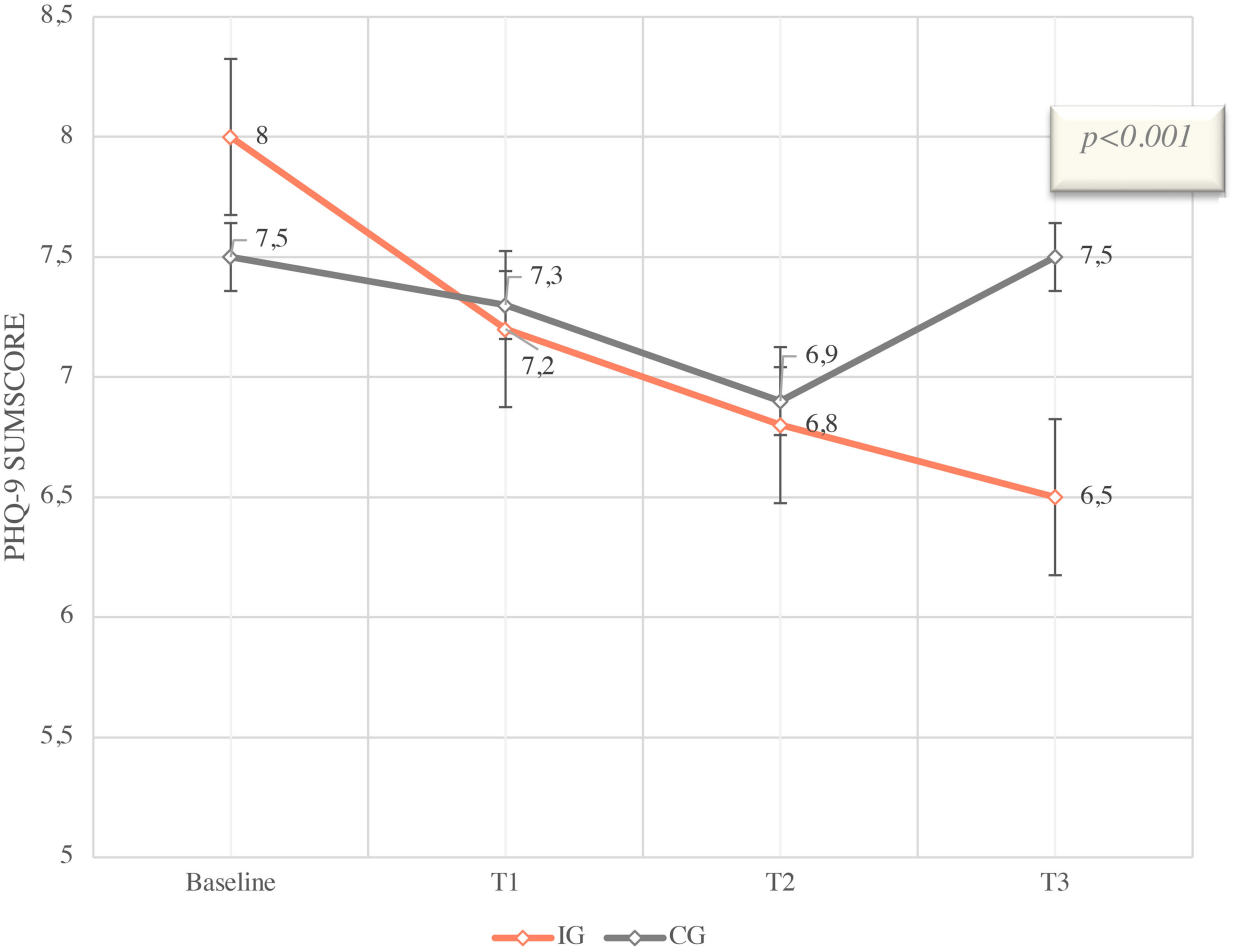
PHQ-9 Sumscore means with SD and 95% CI from Baseline (T0) to T3 (12 weeks), IG vs. CG of ITT colletive (n=396).

A reduction in the total score is associated with a reduction in psychological distress. The reduction of 1.5 score points corresponds to a moderate relative reduction of 18.8% compared to baseline. The use of the app resulted in a significantly higher reduction compared to the control arm [least squares estimate difference between IG and CG -1.52, 95% confidence interval (CI) (1.03; 2.21); p<0.001] in psychological distress shown in **Figure 3**. In the CG, a delta of 0 was measured between baseline and T3. Therefore, no reduction or increase in the PHQ-9 total score, indicating consistent psychological distress, was observed in patients without app usage. The intervention group had a slightly higher baseline value of 8.0 compared to the control group (7.5).

The estimates are based on a mixed model for repeated measurements with group assignment (intervention group versus control group), measurement time, the interaction of group * time, baseline PHQ9 score, and the stratification variable “Therapy/aftercare.” The patient is included as a random effect in the model. Baseline is defined as time point T0. The least squares mean differences between the intervention and control groups are reported.

The effects of the app were also estimated for pre-specified subgroups using the mixed-effects model. The following forest plot displays the results.

### 3.2 PHQ-9 subgroup therapy/aftercare

**Figure 4:**
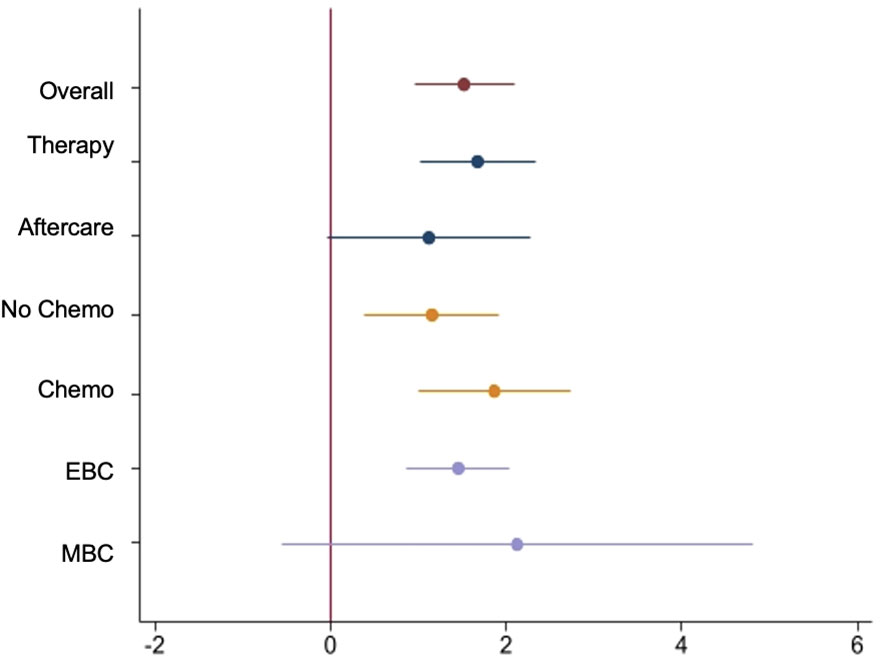
Forest-Plot of Least Squares mean difference of mixed model in overall ITT Set and subgroups.

One subgroup analysis of the primary endpoint considered the time point during the course of therapy. Patients were categorized into the “therapy” group (during acute treatment) or the “aftercare” group. The following **Figure 4** displays the results of the subgroup analysis.

The subgroup “Intervention Therapy” (IG therapy) presents a delta of -1.4 in the PHQ-9 total score from baseline to T3. This corresponds to a relative reduction of 17.3% compared to baseline and a significantly reduced psychological distress during the observation period (least squares estimate difference between IG and CG -1.67, 95% confidence interval (CI) [1,01, 2,33]; p<0.001). The PHQ-9 baseline value in the therapy group is consistent with the initial level of psychological distress in the entire collective. The “Control group Therapy” (CG therapy) exhibits a slightly lower baseline value and a slight increase in psychological distress over the observation period (delta of 0.4 from baseline to T3).

In the “Intervention aftercare” group (IG aftercare), the psychological distress decreases significantly: the delta of -2.1 (least squares estimate difference between IG and CG -1.12, 95% confidence interval (CI) [–0,04, 2,29]; p<0.001) corresponds to a relative reduction of 27.3% in the PHQ-9 total score from baseline to T3. The PHQ-9 baseline value in the IG aftercare is slightly lower than in the overall collective. Thus, the initial psychological distress in the aftercare patients is lower than that of patients in the acute therapy phase. The “Control group aftercare” also shows a slightly lower PHQ-9 baseline value and a delta of -1.1 from baseline to T3.

Results of a mixed model analysis revealed an estimated effect size of 1.67 in IG therapy with a SE of 0.34 and an effect of 1.65 with SE 0.64 in IG aftercare. P-values were p_therapy_<0.001 and p_aftercare_=0.01. Cohen´s d in IG therapy was -0.54 and in IG aftercare -0.44.

### 3.3 PHQ-9 subgroups EBC/MBC

Furthermore, a subgroup analysis was conducted in the “EBC” (early breast cancer) and “MBC” (metastatic breast cancer) groups. This is particularly clinically relevant because in the metastatic setting, psychological distress differs from early breast cancer due to the initial condition of an incurable disease, changes in socioeconomic background, the nature, and especially the duration of treatment, as well as the intensity of distress caused by side effects. Results are displayed in **Figure 4**.

The results show a reduction of -1.5 score points [least squares estimate difference between IG and CG -1.45, 95% confidence interval (CI) (0,87, 2,04); p<0.001] in the PHQ-9 total score in the “Intervention group EBC” (IG EBC). This corresponds to the results of the entire collective. The baseline values of PHQ-9 in EBC compared to the overall collective are only marginally different. The level of psychological distress in the EBC collective is consistent with the average baseline distress in the overall collective. Therefore, the level of psychological distress in the EBC collective is equivalent to the average baseline distress in the intervention group.

On the other hand, the “Intervention group MBC” (IG MBC) exhibits a significantly higher baseline value in the PHQ-9 score compared to the overall collective (9.7 vs. 7.9). The delta from baseline to T3 is -1.3 (least squares estimate difference between IG and CG -2.13, 95% confidence interval (CI) [–0,56, 4,82]; p=0.12), corresponding to a relative reduction of 13.4% in the PHQ-9 total score compared to baseline.

The “Control group” in EBC shows a delta of -0.1 from baseline to T3. The “Control group MBC” (CG MBC) however, shows an increase of 0.6 points in the PHQ-9 total score over the observation period. The baseline values of the control groups EBC and MBC differ. The number of MBC patients was n=9 in the intervention group and n=17 in the control group.

### 3.4 PHQ-9 subgroups CHT/nCHT

The subgroup “Intervention with chemotherapy” (IG CHT) shows a delta of -1.9 score points from baseline to T3 [least squares estimate difference between IG and CG -1.87, 95% confidence interval (CI) (1,00, 2,7); p<0.001]. The delta, representing the reduction in psychological distress, is even more pronounced in patients undergoing chemotherapy compared to the overall IG (**Figure 2**). This corresponds to a relative reduction of 22.1% in the PHQ-9 total score compared to baseline. Additionally, the PHQ-9 baseline value in the “Intervention with chemotherapy” subgroup is higher than the average baseline value of the entire collective. The “Control group with chemotherapy” (CG CHT) shows a delta of 0.3 from baseline to T3, indicating consistent psychological distress over the 12-week observation period. The PHQ-9 baseline value is also higher than in the overall collective.

The “Intervention group without chemotherapy” (IG nCHT) shows a delta of -1.3 score points from baseline to T3 (least squares estimate difference between IG and CG -1.15, 95% confidence interval (CI) [0,39, 1,92]; p=0.003). The effect of the app on reducing psychological distress is slightly smaller in patients without chemotherapy compared to those undergoing chemotherapy or the overall collective. The baseline value of 7.5 is also slightly lower than that of the overall intervention group. The “Control group without chemotherapy” (CG nCHT) shows a delta of -0.2 in the PHQ-9 total score from baseline to T3, indicating almost consistent psychological distress. The PHQ-9 baseline value of 7.2 is slightly lower than the value of the overall control group (7.5) and also slightly lower than the value of the intervention group (8.0).

Results of a mixed model analysis revealed an estimated effect size of 1.87 in IG CHT with a SE of 0.44 and an effect of 1.15 with SE 0.39 in the IG nCHT subgroup.

47 out of a total of 191 ITT patients in the intervention group showed a usage time of 0 minutes after 12 weeks. This means that approximately 25% of the patients did not use the app throughout the entire intervention period.

The remaining patients had an average usage time of 466.4 minutes in the first month (baseline to T1), equivalent to around 17 minutes per day. In the second month from T1 to T2, the usage time was 274 minutes, or 10 minutes per day. In the third month from T2 to T3, it was 313 minutes. It appears that about a quarter of the patients did not use the app or used it only briefly. The remaining 75% used the app intensively and throughout the entire intervention period and all elements of the app.

## 4 Discussion

This multicenter, randomized controlled study with a waitlist control aimed to primarily investigate whether the psychological distress caused by a breast cancer diagnosis, one of the most relevant side effects during treatment and aftercare, could be improved through an app-based coaching program (PINK! Coach).

The study population was representative and adequately reflected the diversity of diagnosis and treatment situations, including factors such as age, tumor stages, and tumor biological characteristics.

The average age of breast cancer diagnosis is approximately 64 years (7), with one in four affected individuals being younger than 55 years and one in ten being younger than 45 years old. The median age of the patients in the population studied in this study was 52 years, which is lower than the average age of the general breast cancer population. This can be attributed to the fact that the participating study centers are clinically and scientifically specialized certified breast centers located in larger cities, which also offer specific treatment options for younger patients. These centers have an interdisciplinary diagnostic, therapeutic, and supportive network in place to address issues such as fertility preservation, genetic predisposition, etc., and incorporate them directly into the optimal treatment plan. Younger patients are therefore deliberately referred to such centers. The willingness to participate in a clinical study can also be age-dependent. However, the study also included numerous older patients who actively and consistently utilized the intervention throughout the study period.

Approximately 20% of women with breast cancer experience tumor spread and the formation of metastases despite treatment, occurring over months to years. About 7% of women are diagnosed with metastases at the time of their initial breast cancer diagnosis (primary metastatic patients).

In this study, only MBC patients with newly diagnosed metastasis were included. The study population comprises 7% MBC patients, which adequately reflects the clinical reality. The distribution of MBC patients between the intervention and control groups is similar in the full analysis set (ITT).

Regarding tumor biological characteristics (TNM, TNBC/HR+/HER2+/-), the studied population is representative. For example, approximately 15-20% of breast cancer tumors are HER2-positive. In the studied population, 20.2% of patients had a HER2-positive tumor, which aligns with the real-world population of breast cancer patients. In the past, HER2-positive breast cancer tumors were considered as an aggressive tumor type with a poor prognosis. However, the introduction of targeted therapies in form of HER2-directed drugs, has significantly improved therapeutic options (7). From being a negative prognostic factor, HER2 positivity has become a positive predictive factor, indicating response to targeted anti-HER2 therapies. Therefore, patients with HER2-positive tumors now have a good prognosis due to the available therapeutic options. However, the use of anti-HER2 therapies is often associated with substantial side effects, which has implications for psychological distress. In addition, the treatment duration with anti-HER2 therapies is significantly longer, which adds to the burden on patients (7).

The distribution of patients with triple-negative breast cancer (TNBC) and hormone receptor-positive tumors (ER and/or PR positive) is approximately equal between the two study groups (intervention vs. control). The proportion of TNBC patients in the studied population is 20.8%. This proportion aligns with the expected occurrence of TNBCs in this study. The slightly higher percentage and the younger age of the population may be influenced by the nature of the study center. Patients with TNBC are often younger, more willing to travel longer distances to a specialized center and are frequently enrolled in clinical trials. Patients with TNBC have a high psycho-oncological support need because achieving a non-PCR (Pathological Complete Remission) is more common compared to other types of breast cancer. Consequently, treatment approaches often need to be escalated, leading to a longer duration of treatment. Additionally, TNBC is more frequently diagnosed in younger patients, and they often experience greater psychological distress due to their family situation. TNBC is also often associated with genetic factors, which can further increase the burden. Overall, the psychological impact of TNBC can be significant, requiring specialized support and care to address the emotional and mental well-being of patients facing this challenging form of breast cancer (7, 32–34).

The primary endpoint of psychological distress was measured using the validated PHQ-9 questionnaire. The PHQ-9 questionnaire is a commonly used tool for assessing depressive symptoms. It consists of nine questions based on the nine diagnostic criteria for a depressive disorder according to the Diagnostic and Statistical Manual of Mental Disorders (DSM-5) (31).

In the overall full analysis set (FAS) population, a reduction of -1.5 score points (least squares estimate difference between IG and CG -1.52; 95% confidence interval (CI) [–1,91, –1,07] in the PHQ-9 was observed in the intervention group. The estimates are based on a mixed model for repeated measurements with group assignment (intervention group versus control group), measurement time, the interaction of group * time, baseline PHQ9 score, and the stratification variable “Therapy/aftercare.” The patient was included as a random effect in the model. Baseline is defined as time point T0. The least squares mean differences between the intervention and control groups were reported. This corresponds to a significant and clinically meaningful reduction (p<0.001) compared to the control group.

In the control group, there was no change in the PHQ-9 from baseline to T3. A reduction of 1.5 points in the PHQ-9 score can be interpreted as a slight improvement in depressive symptoms. It is important to note that the clinical relevance of such a change can vary individually and depends on other factors, such as the severity of baseline symptoms, individual patient perception, and daily functioning. In the intervention group, at baseline, 61 out of 170 patients, or 36%, had a PHQ-9 score above 10, indicating moderate to severe depression (31–33).

In clinical studies or research work, statistical methods such as effect estimations from mixed models can be used to assess the clinical relevance of observed changes. In the FAS population, an effect size of 1.52 was found. A reduction of 1.5 points in the PHQ-9 score and an estimated effect size of 1.52 of the mixed model can be considered as a moderate effect size. This result is further supported by the calculated effect size according to Cohen, which is d=0.5 for the entire cohort.

Other studies, even with other DiGAs with psychological endpoints, show similar results in sumscore reduction and cohen´s d (35–37). However, the clinical significance should always be considered in the context of the individual situation and the overall symptomatology. Patients often subjectively perceive the improvement in their mental well-being as highly relevant (9, 10, 33).

Studies that have examined the PHQ-9 questionnaire in breast cancer patients, the extent of depressive symptoms, and the relationship between breast cancer and mental health have provided two important insights: breast cancer patients have an increased prevalence of depression. Studies have shown that breast cancer patients may have an increased risk of experiencing depressive symptoms and depression. The prevalence varies depending on the study, but some studies report rates as high as 40% (compared to 36% in the population).

Furthermore, there is a correlation between psychological distress and different treatment phases as well as the age of breast cancer patients. Various treatment phases such as diagnosis, surgery, chemotherapy, or aftercare care can be associated with an increased risk of depressive symptoms in breast cancer patients.

The data from this study show a higher baseline value of PHQ-9 in the subgroup receiving chemotherapy, as well as a larger reduction over the 12-week period. In comparison, the control group showed no change in the PHQ-9. The subgroup without chemotherapy, however, had a lower baseline value and a slightly smaller delta over 12 weeks. Nevertheless, the data from both subgroups were significant (p<0.001) when compared between intervention and control. From a clinical perspective, these results are expected because chemotherapy for the treatment of breast cancer can cause various psychological side effects. These effects can vary individually and depend on several factors such as the type of chemotherapy, dosage, individual tolerability, the patient’s psychological condition before treatment, as well as the fact that chemotherapy is indicated – which can trigger anxiety and concerns. However, the data also show that patients who did not receive chemotherapy still experience a similar level of psychological distress as patients who underwent chemotherapy. A cancer diagnosis itself, regardless of the stage and tumor biological characteristics, can profoundly impact one’s life.

The intervention with PINK! Coach had significant effects on both groups, which are clinically meaningful. The correct clinical interpretation of alterations on a numerical scale should take into account not just statistical significance but also whether the observed change holds significance for patients. Equivalent changes on a numerical scale might carry distinct clinical importance in various patient populations, such as those differing in age, disease severity, or injury type. Additionally, statistical significance is intertwined with the sample size.

To assess the clinical relevance of the determined results, an attempt was made to determine the Minimal Clinically Important Difference (MCID) in addition to calculating effect sizes using Cohen’s method. The Minimal Clinically Important Difference of the PHQ-9 has not yet been determined for a cohort of breast cancer patients. There are no publications on this topic in the known databases. However, there are publications on the MCID of the PHQ-9 in other patient cohorts with different diagnoses. For determining clinical relevance there is currently no established methodological approach. Additionally, there are currently no comparative data from other publications that have investigated the same population with a comparable intervention. Consequently, more research is needed to contextualize the collected data.

In recent years, research has focused on the development of personalized treatment concepts. Therapeutic decisions are mainly based on molecular and histological characteristics of the tumor. The primary goal is to find optimal treatment pathways or tailor treatments specifically to early breast cancer patients (EBC), taking into account long-term toxic side effects and improving quality of life. This has led to a significant de-escalation of therapy and a lower rate of chemotherapy being performed, as observed in the studied population. However, the data also confirm that even without chemotherapy, breast cancer patients experience a high level of psychological distress (38).

Both EBC and MBC patients experience psychological and social distress after diagnosis, during treatment, and in the follow-up period one year after diagnosis. This was confirmed by the data available. The ongoing follow-up (at 6 and 12 months) will provide a more detailed insight.

Another subgroup analysis of the population, divided into “therapy” and “aftercare,” reveals similar results. The subgroup “intervention therapy” shows a delta of -1.5 from baseline to T3, which corresponds to a relative reduction of 18.3% compared to baseline. The baseline value is consistent with that of the entire population. The “control group therapy” has a lower baseline value and a delta of 0.3 from baseline to T3, indicating a slight increase in psychological distress. The “intervention aftercare” group shows a delta of -2.1, corresponding to a relative reduction of 34.6% from baseline to T3. For comparison, a two-year treatment with the medication Abemaciclib in patients with hormone receptor-positive high-risk early-stage carcinoma results in a relative risk reduction of 35% in terms of invasive recurrence or distant metastasis - which is generally considered a clinically significant effect. However, comprehensive therapy includes not only efficacy in treating the tumor but also physical and psychological well-being.

The baseline value is lower than that of the entire population. The “control group aftercare” also shows a slightly lower baseline value and a delta of -1.1 from baseline to T3. It was also observed that the intervention in both subgroups led to a significant reduction in psychological distress compared to the control group. These results indicate that the intensity of psychological distress is highly individual but can still be reduced through clinically significant interventions.

The study has reached its primary endpoint after 12 weeks of intervention. The comparison between study groups in the overall population and subgroups are significant. The studied population is comparable to the entire population of breast cancer patients, indicating that PINK! Coach helps all affected individuals in terms of psychological distress. The study data suggests that PINK! Coach can be established as a routine component of breast cancer care and is also accepted by the patients as part of their therapy and/or aftercare.

PINK! Coach is structured in a multimodal manner and provides patients with personalized content based on their information regarding therapy, tumor biology, age, and personal situation. Patients receive daily tasks aimed at sustainably improving their lifestyle regarding nutrition, physical activity and mental health. This approach is designed to ensure ongoing motivation and empowerment. In terms of results, especially within subgroups, this personalization appears to help many patients in different situations to change their lifestyle. As the emergence of psychological distress is highly individual and complex, it is reasonable to assume that the reduction of this mental burden is also complex and attributable to various functions of the app.

Customization of content and coaching proves effective for all patients across diverse subgroups in a disease as heterogeneous as breast cancer. Educational content reduces fears and enhances self-management, empowering patients to actively participate in their therapy (27–29, 32, 33, 39). Nevertheless, more research is necessary to understand the exact mechanism of action of the app.

The data on dropouts from the study indicate that patients who discontinued their participation mainly did so because they were randomized into the control group but desired immediate use of the app. This highlights the high demand for low-threshold support options that can be easily integrated into daily life and routines. This suggests that the implementation of PINK! Coach in breast cancer care is a promising addition to therapy management and patient support.

Utilization of mobile health (mHealth) applications within clinical environments is increasing. mHealth apps have been employed to enhance preventive measures, enhance early detection, facilitate care management, and provide assistance to both survivors and individuals dealing with chronic conditions. Nevertheless, there exists a scarcity of comprehensive information regarding the effectiveness and practicality of these mHealth apps (40). Considering the increasing number of mHealth apps available to patients and their increasing use in breast cancer care, it is important to understand their effects. Different international studies (26) of the last years showed promising results in patient-doctor communication, therapy management, health-related quality of life, BMI reduction and increasing physical activity as well as stress reduction (41, 42). Nevertheless, there is a lack of compelling data regarding the advantages of mHealth in addressing persistent adverse psychological effects (43). Therefore, long-term data with breast cancer survivors are necessary to investigate whether the effects observed so far endure. To generate long-term data on the positive psychological effects of the PINK! app, a 1-year follow-up has been planned, which will provide insight into the extent to which the observed effects persist.

Nevertheless, mHealth applications hold substantial significance for both developed nations and emerging economies, offering an economical means to extend healthcare access and deliver health-related information on a broader scale (40). In Germany, the highest proportion of patients receiving psycho-oncological care are breast cancer patients (66.7%) (44). With the increasing number of cases and the rising demand for psycho-oncological support for breast cancer patients and survivors, the healthcare system is reaching its capacity limits. The resources at clinics are no longer sufficient to accommodate such a high number of patients requiring long-term psycho-oncological care. As a result, evidence-based mHealth apps aimed at reducing psychological distress also hold significant potential in Germany (22, 30, 45).

## 5 Conclusion

By personalizing its content, PINK! Coach empowers patients to positively influence their own lifestyle. The individual situation and needs of the patients are taken into account. This allows patients to set individual priorities and engage with aspects that are important to them, impacting their quality of life and mental well-being. The factors influencing the quality of life and mental health of patients in the context of breast cancer diagnosis and treatment are diverse. This is consistent with our observation that various subgroups experience significant benefits from using PINK! Coach.

## Data availability statement

The raw data supporting the conclusions of this article will be made available by the authors, without undue reservation.

## Ethics statement

The studies involving humans were approved by Medical Ethical Committee of the LMU University of Munich, Germany on 21.07.2022 (Reference number: 22-0498). The studies were conducted in accordance with the local legislation and institutional requirements. The participants provided their written informed consent to participate in this study.

## Author contributions

JW: Conceptualization, Formal analysis, Methodology, Visualization, Writing – original draft, Writing – review & editing. SvS: Data curation, Project administration, Software, Writing – review & editing. PW: Conceptualization, Funding acquisition, Methodology, Resources, Supervision, Writing – review & editing. ML: Conceptualization, Investigation, Methodology, Writing – review & editing. CzE: Conceptualization, Data curation, Methodology, Validation, Writing – review & editing. MS: Conceptualization, Methodology, Validation, Writing – review & editing. FB: Conceptualization, Methodology, Writing – review & editing. StS: Conceptualization, Investigation, Writing – review & editing. SK: Investigation, Writing – review & editing. MT: Investigation, Writing – review & editing. JT: Investigation, Writing – review & editing. MB: Investigation, Writing – review & editing. HH: Investigation, Methodology, Writing – review & editing. AS: Project administration, Writing – review & editing. FH: Project administration, Writing – review & editing. NH: Conceptualization, Methodology, Supervision, Writing – review & editing. RW: Conceptualization, Formal analysis, Investigation, Methodology, Project administration, Supervision, Validation, Writing – review & editing, Writing – original draft.

## Footnotes

1
https://www.psychotherapeutenkammer-berlin.de/ 26.07.2023.

2
https://diga.bfarm.de/de/verzeichnis 29.06.2023.
